# Supplementary material for: Multidrug-resistant and carbapenemase-producing critical gram-negative bacteria isolated from the intensive care unit environment in Amhara region, Ethiopia
Source: PLoS One. 2023 Nov 30;18(11):e0295286. doi: 10.1371/journal.pone.0295286 (PMC10688904; doi:10.1371/journal.pone.0295286)
Supplement: Attachment — Submitted filename: Response to reviewers.docx [file pone.0295286.s001.docx]

**Response to reviewers**

Response to the academic editor

1. Please ensure that your manuscript meets PLOS ONE's style requirements, including those for file naming. The PLOS ONE style templates can be found at 
https://journals.plos.org/plosone/s/file?id=wjVg/PLOSOne_formatting_sample_main_body.pdf and 
https://journals.plos.org/plosone/s/file?id=ba62/PLOSOne_formatting_sample_title_authors_affiliations.pdf

Response: all the formatting for headings, tables, reference brackets in the body, etc… are corrected according to the journal style and all highlighted in the manuscript

2. We suggest you thoroughly copyedit your manuscript for language usage, spelling, and grammar. If you do not know anyone who can help you do this, you may wish to consider employing a professional scientific editing service.

Whilst you may use any professional scientific editing service of your choice, PLOS has partnered with both American Journal Experts (AJE) and Editage to provide discounted services to PLOS authors. Both organizations have experience helping authors meet PLOS guidelines and can provide language editing, translation, manuscript formatting, and figure formatting to ensure your manuscript meets our submission guidelines. To take advantage of our partnership with AJE, visit the AJE website (http://learn.aje.com/plos/) for a 15% discount off AJE services. To take advantage of our partnership with Editage, visit the Editage website (www.editage.com) and enter referral code PLOSEDIT for a 15% discount off Editage services.  If the PLOS editorial team finds any language issues in text that either AJE or Editage has edited, the service provider will re-edit the text for free.

Upon resubmission, please provide the following: 
● The name of the colleague or the details of the professional service that edited your manuscript
● A copy of your manuscript showing your changes by either highlighting them or using track changes (uploaded as a *supporting information* file)
● A clean copy of the edited manuscript (uploaded as the new *manuscript* file)

Response: Edited and attached as suggested

The manuscript is edited by Solomon Taye Sima, PhD; Postdoctoral Research Scientist; Center for Immunity and Inflammation; New Jersey Medical School; Rutgers University.

3. In your Data Availability statement, you have not specified where the minimal data set underlying the results described in your manuscript can be found. PLOS defines a study's minimal data set as the underlying data used to reach the conclusions drawn in the manuscript and any additional data required to replicate the reported study findings in their entirety. All PLOS journals require that the minimal data set be made fully available. For more information about our data policy, please see http://journals.plos.org/plosone/s/data-availability.

"Upon re-submitting your revised manuscript, please upload your study’s minimal underlying data set as either Supporting Information files or to a stable, public repository and include the relevant URLs, DOIs, or accession numbers within your revised cover letter. For a list of acceptable repositories, please see http://journals.plos.org/plosone/s/data-availability#loc-recommended-repositories. Any potentially identifying patient information must be fully anonymized.

Important: If there are ethical or legal restrictions to sharing your data publicly, please explain these restrictions in detail. Please see our guidelines for more information on what we consider unacceptable restrictions to publicly sharing data: http://journals.plos.org/plosone/s/data-availability#loc-unacceptable-data-access-restrictions. Note that it is not acceptable for the authors to be the sole named individuals responsible for ensuring data access.

We will update your Data Availability statement to reflect the information you provide in your cover letter.

Response: All relevant data are within the manuscript.

4. PLOS requires an ORCID iD for the corresponding author in Editorial Manager on papers submitted after December 6th, 2016. Please ensure that you have an ORCID iD and that it is validated in Editorial Manager. To do this, go to ‘Update my Information’ (in the upper left-hand corner of the main menu), and click on the Fetch/Validate link next to the ORCID field. This will take you to the ORCID site and allow you to create a new iD or authenticate a pre-existing iD in Editorial Manager. Please see the following video for instructions on linking an ORCID iD to your Editorial Manager account: <https://www.youtube.com/watch?v=_xcclfuvtxQ>

A pre-existing iD in Editorial Manager is authenticated

5. Please include your full ethics statement in the ‘Methods’ section of your manuscript file. In your statement, please include the full name of the IRB or ethics committee who approved or waived your study, as well as whether or not you obtained informed written or verbal consent. If consent was waived for your study, please include this information in your statement as well.

Response: corrected as suggested and highlighted in lines 118-121

Additional Editor Comments:

Response to additional editor comments

Line 22: ‘amied’
Comment: typo
Response: corrected and highlighted in line 22

Lines 23-24: “……..gram negative bacteria (K. pneumoniae, E. coli, P. aeruginosa and Acinetobacter species) and their antibiotic resistance on ICU environmental surfaces”
Comment: Slightly modify the sentence “……..Gram negative bacteria (Klebsiella pneumoniae, Escherichia coli, Pseudomonas aeruginosa and Acinetobacter species) on ICU environmental surfaces and their antibiotic resistance……….”

Response: corrected as suggested and indicated in lines 23-24

Lines 46-47: “Moreover, the majority of carbapenemase-encoding genes are carried by mobile elements, such as plasmids, which will ease the vertical transmission of resistance across other GNB in the same setting”
Comment: In context of mobile genetic elements, probably the authors wanted to mean horizontal transmission of resistance determinants but wrote ‘vertical transmission’.

Response: corrected and highlighted in line 47

Line 73: “High-touch surfaces are those with frequent contact with the hands, which poses the greatest risk of transmission of microorganisms”
Comment: It will be better to mention that the authors targeted high-touch surfaces for environmental sampling before defining high-touch surfaces.

Response: corrected as suggested

Line 74: ‘bed masters’
Comment: Probable typo

Response: corrected and highlighted in line 73

Line 82: “Single distinct colonies were isolated and purified by subculturing in fresh BAP medium to obtain pure culture isolates”
Comment: Authors should explain how they have chosen single distinct colonies for sub-culture. Environmental samples are likely to yield multiple organisms. Have authors considered culturing all types of isolates?

Response: Thank you for the comment. Single distinct colonies means these colonies preliminary differentiated based on morphology on media. Morphologically deferent means that difference in size, shape, edge, lactose fermentation, mucoid or dry etc… Then, biochemical test was done for all morphologically deferent isolates.

Lines 83-84: “Isolates were identified using different biochemical tests such as triple sugar iron agar, indole, motility, urease production, hydrogen sulfide production, citrate utilization, lysine decarboxylase tests, and oxidase strip tests”
Comment: Mentioned tests may not be enough to correctly identify Acinetobacter spp. Please explain.

Response: I really appreciate the comment. But, through the editing process, an additional test, the catalase test, was deleted from the list of mentioned tests.

In this study, most of the Acinetobacter colonies on MacConkey agar were non-lactose fermenters, with raised and smooth-edged colonies, but very few were sticky and flat. In addition to colony morphology characteristics, the above-mentioned biochemical tests, catalase and oxidase strip tests, were used to differentiate Acinetobacter spp.

Line 122: “A total of 384 environmental samples from neonates (246/384=64.1%) and other intensive care units (150/384=35.9%) were analyzed”.
Comment: Please check the statistics mentioned in the line.

Response: I accept the commented and highlighted in line 134

Line 123: “Of 126(32.8%) culture positive samples, 162 critical GNB isolates……”
Comment: Write “From 126(32.8%) culture positive samples, 162 critical GNB isolates….”

Response: corrected as suggested and highlighted in lines 135-136

Line 166: “Moreover, the magnitudes of MDR critical GNB were 75/95(78.9%) and 53/67(80.5%) at FHSCH, respectively”
Comment: Please check the above sentence. It seems incomplete.

Response: corrected and highlighted in line 165

Lines 166-168: “The neonatal intensive care unit of UoGCSH was more contaminated by MDR and CP-critical GNB than other ICUs. In contrast, at the FHCSH, the ICUs for pediatric, adult, and surgical critical care patients were more contaminated than the neonatal units”.
Comment: Just based on the proportions, it will not be prudent to mention on the level of contamination of different areas without doing relevant statistical analysis.

Response: I really accept the comment. But, when we did the analysis, the contamination rate among units was not statistically significant. Therefore, I corrected the statement and indicated in lines 165-168

Lines 191-192: CP K. pneumoniae isolates were 25-87.5% resistant to amikacin, ciprofloxacin, chloramphenicol, piperacillin-tazobactam, gentamicin, sulfamethoxazole-trimethoprim and tetracycline, respectively.
Comment: It is difficult to follow the actual magnitude of resistance to different antibiotics from the above sentences. Authors are advised to either write to refer to Table 5 or mention resistance rates of all antibiotics individually.

Response: I accept the comment and the corrected sentence highlighted in lines 186-187 and 189-190
Lines 283: “LMIC’
Comment: Please expand ‘LMIC’ where it is first used.

Response: corrected and highlighted in line 257

Table 2: ‘R’ refers to resistant to classes of antibiotics as per the footnote. But in reality, authors have considered types of antibiotics for calculation of ‘R1 to R15’ not antibiotic classes. Few statistics are little bit misleading because not all drugs were tested for all organisms. Therefore, the higher values of R like ‘R15’ should be denoted as ‘-‘ not as ‘zero’.

Response: Thank you for valuable comment. The table is amended as it was mentioned in the footnote and highlighted in the table
Table 3: Please delete the number of samples collected from the second row.

Response: corrected as suggested
Table 4: The column totals of few columns are not matching with what mentioned in the table. Please check

Response: corrected and highlighted in the table

Overall comment: Please mention the strengths and limitation of the present study toward the end of the Discussion.

Response: corrected and highlighted in lines 262-266

Please also mention the measures which are planned to control environmental contamination with critical Gram-negative bacteria at the study centers.

Response: Thank you for the comment. As researcher, we already informed concerned bodies (head of hospitals) of two sites to take actions.

Review Comments to the Author

Reviewer #1: 

1. Please define MDR in the manuscript (resistance to how many antibiotics)

Response: I accept the comment. It is added in manuscript and highlighted in line 93

2. The samples have grown only gram negative organisms. No gram positive (S. aureus/ CONS) have been reported in any of the samples collected. This is in contrast to that seen in most other studies such as Darge, A., Kahsay, A.G., Hailekiros, H. et al. Bacterial contamination and antimicrobial susceptibility patterns of intensive care units medical equipment and inanimate surfaces at Ayder Comprehensive Specialized Hospital, Mekelle, Northern Ethiopia. BMC Res Notes 12, 621 (2019).

Response: I really appreciate the comment given by the reviewer; however, my objective was only on the critical GNB as mentioned in the manuscript.
3. It would be more useful if in the discussion the authors could give a table of the observations of the other similar studies. This would help identify the local epidemiology of the pathogens based on the regions

Response: Thank you for the comment. But, as per my search similar studies have been already discussed.
4. It would be useful if the authors could add if any of the patients in the ICU developed infection with these organisms. A detailed profile of the infective pathogens and the infections caused in the ICU patients during this timeframe would help understand the relevance of these isolates.

Response: Thank you for valuable comment. However, this paper is one part of my phd works. The final objective of this work was to see the transmission link or to detect the sources of infection for carbapenemase producing critical GNB in ICUs. Therefore, I collected environmental, clinical and rectal swab samples from ICUs. However, due to resource limitation for gene detection and whole genome sequencing other works were not yet finished.

Reviewer #2:

1. The terminology 'healthcare-associated infection' instead of 'hospital acquired infection' would be better suited here. Please make similar changes wherever applicable throughout the manuscript.

Response: corrected as suggested throughout the manuscript

2. Sampling technique: Did the number of environmental sample collection took place at random, or any criteria (like sample size calculation) for sample collection was used? Please clarify in the main manuscript.

Response: I accept the comment. It is clarified and highlighted in line 71

3. The acronym "BAP" appears in line no. 82, I imagine that the authors refer to: "blood agar plates." elaborate the acronym "BAP” since I did not find the acronym in another part of the text.

Response: I accept the comment. It is elaborated in line 80

4. In antibiotic susceptibility result by disk diffusion method the authors have not mentioned anything about the intermediate results in the isolates. Were they counted as resistant or susceptible?

Response: I accept the comment. Critical GNB isolates with intermediate results detected were very few in numbers, however, for analysis counted as resistant.

5. sCIM method for testing carbapenemase production in A. baumannii is not recommended by standard guidelines. Hence, few isolates should also be validated for the same by some other methods.

Response: I really appreciate comment given by the reviewer. However, due to lack of resource confirmatory molecular test was not done and this is indicated as limitation of this study

6. There are spacing errors in the text throughout the manuscript that should be corrected.

Response: corrected as suggested
