# Supplementary material for: Multidrug-resistant and carbapenemase-producing critical gram-negative bacteria isolated from the intensive care unit environment in Amhara region, Ethiopia
Source: PLoS One. 2023 Nov 30;18(11):e0295286. doi: 10.1371/journal.pone.0295286 (PMC10688904; doi:10.1371/journal.pone.0295286)
Supplement: Attachment — Submitted filename: Response to Reviewers.docx [file pone.0295286.s002.docx]

**Response to reviewers**

**Journal requirements:**

Please review your reference list to ensure that it is complete and correct. If you have cited papers that have been retracted, please include the rationale for doing so in the manuscript text, or remove these references and replace them with relevant current references. Any changes to the reference list should be mentioned in the rebuttal letter that accompanies your revised manuscript. If you need to cite a retracted article, indicate the article’s retracted status in the References list and also include a citation and full reference for the retraction notice.

**Author response:** It is complete and corrected in the document

**Author response to academic editor**

**Editor comments**: #Authors do not explicitly mention the specific study design used in the research.

**Author response:** Thank you for the comment. It is corrected and included in line 72.

**Editor comment**:-# The following reviewers' comment has not been addressed correctly. Line 73: “High-touch surfaces are those with frequent contact with the hands, which poses the greatest risk of transmission of microorganisms”. Comment: It will be better to mention that the authors targeted high-touch surfaces for
environmental sampling before defining high-touch surfaces.

**Author response:** Thank you for the comment. I mention the targeted high-touch surfaces in the study before the definition. Line 72-75 of the document.

**Editor comment:**#Line 120:The "Registration number" should be accessible by a valid link.

**Author response:** In our institution there is no online access for registration number to the ethical letter. The institution simply gives the hardcopy letter for the researchers. This document is already attached to PLOS ONE during submission.

**Editor comment**: #Although the information presented in Table 2 is interesting, such a classification is not familiar in the field of clinical microbiology, so I recommend that you present it based on standard interpretations instead of such an explanation:
pan-sensitive, MDRو XDR, and PDR
**Authors Response:**Thank you for the valuable comment. The focus of the current study is on MDR and carbapenemase producing isolates (already included in Table 2). On the other hand, according to the international experts to say XDR (nonsusceptibility to at least one agent in all but two or fewer antimicrobial categories i.e., bacterial isolates remain susceptible to only one or two categories) and PDR (nonsusceptibility to all agents in all antimicrobial categories) bacterial isolates should be tested against all or nearly all of the antimicrobial agents within the antimicrobial categories.

(<https://www.sciencedirect.com/science/article/pii/S1198743X14616323>)

The current study was not able to say PDR since the isolates were not tested to all of the antimicrobialagents within the antimicrobial categories listed by international experts such as aztreonam, fosfomycin and colistin.

Moreover, the table 2 was prepared by looking recent published papers in PLOS ONE journal (<https://doi.org/10.1371/journal.pone.0264818> and <https://doi.org/10.1371/journal.pone.0256556.t002>), used R0, R1….

Finally, if I have to remove the columns for R0-R11, I am willing to do so.

**Author response to the reviewers**

**Review Comments to the Author**

**Reviewer comment**#1: I appreciate the work done by the team and recommend acceptance of the manuscript. I recommend that the authors should publish the clinical data as well

**Author response:** Thank you for all the valuable comments. I will send clinical data manuscript for publication the as soon as possible.

**Reviewer comment** #2: Most of the issues raised were successfully addressed by the authors. A few still need to be further clarified:
Line 18. Insert (HAI) after healthcare-associated infections and then the abbreviation can be used throughout the abstract.

**Author response:** Thank you for the comment. It is corrected as suggested and indicated in line 18,19 and 20.

**Reviewer comment**: Line 66. Owing to innovation or renovation?

**Author response:** It is renovation and corrected in line 66.

**Reviewer comment**: Line 71. Better to use, samples from the hospital environment instead of environmental samples. Please do similar corrections wherever applicable

**Author response:** It is corrected as suggested throughout the document.

**Reviewer comment** Line 92. I think second generation cephalosporins are also comes under extended-spectrum cephalosporins if so then, either write first & second generation cephalosporins or remove ‘non-extended cephalosporins’ or cefuroxime might be added to the extended-spectrum cephalosporins category in the above line 89-90

**Author response:** It is corrected by removing non-extended and extended cephalosporin replacing with first and second, and third and fourth generation cephalosporins, respectively. It is highlighted in line 90, 91, 93 and in table-1 in the main document.

**Reviewer comment**: Line 136. ‘162 critical GNB isolates were identified and 36 samples showed mixed growth. Add the percentage for these two results also

**Authors response:** corrected as suggested and included in line138

**Reviewer comment**: Line 152-153. ‘The most common MDR isolate was *K. pneumoniae*’. Remove this line, as the next statement is enough to explain that the K. pneumoniae was the most common MDR organism.

**Author response:** corrected as suggested

**Reviewer comment:** Line 177-178. I think it would be better to write, two different CP critical GNB rather than two types of CP critical GNB as its confusing

**Authors response:** corrected as suggested (line 178)

**Reviewer comment**:-Figure is of low resolution. Please endeavour to get a high quality figure which again can be changed in size without losing quality/resolution.

**Author response:** corrected as suggested

**Reviewer comment**:-Some minor grammatical as well as typographical errors are still present in the manuscript that needs to be corrected. I’m pointing a few as follows-
Line 18. Insert ‘are’ between ‘infections’ and ‘common’

**Authors response:** corrected as suggested in line 18

**Reviewer comment**:-Line 31. Change to ‘were detected on most of the surfaces from the hospital environment’

**Authors response:** corrected as suggested in line 31 and 32

**Reviewer comment**:-Line 32. Insert ‘from the’ between ‘especially’ and ‘baby bed sets and incubators’.

**Authors response:** corrected as suggested in line 32

**Reviewer comment:-**Line 34. Correct carpapenemase

**Author response:** corrected as suggested in line 34

**Reviewer comment** Line 47. Replace ‘will’ with ‘might’ ease the horizontal transmission of

**Author response:** corrected as suggested in line 47

**Reviewer comment**: Line 80. use ‘into’ instead of ‘in’ before subculturing

**Author response:** corrected as suggested in line 81

**Reviewer comment**:-Line 99 & 104. Correct 0.5McFarl to 0.5 McFarland

**Authors response:** corrected as suggested in line 101 and 106

**Reviewer comment**:-Line 121. Insert ‘of the’ between ‘environmental surface’ ‘ICUs’.

**Authors response:** corrected as suggested in line 123

**Reviewer comment**: Line 151-152. Remove ‘of’ after 128(79 %)

**Author response:** corrected as suggested
